# The effects of materialism and ego depletion on intertemporal choice: An event-related potential study

**DOI:** 10.3389/fpsyg.2022.1051405

**Published:** 2022-12-06

**Authors:** Yingying Pei, Junjian Yu, Lijun Zhao

**Affiliations:** ^1^Department of Psychology, School of Education Science, Liaocheng University, Liaocheng, China; ^2^School of Psychology, Shandong Normal University, Jinan, China

**Keywords:** intertemporal choice, materialism, ego depletion, situation, self-control

## Abstract

**Objective:**

The study aims to promote human beings to make scientific and reasonable decisions for the long-term and beautiful future.

**Methods:**

We designed two experiments to explore the influence of materialism and ego depletion from the perspective of behavioral decision-making and neural mechanism.

**Results:**

In Experiment 1, there was asymmetry in intertemporal choice between gain and loss situations. In the gain situation, high materialism were more likely to choose the later and larger option (LL). However, in a loss situation, we found a reverse sign effect, and the proportion of subjects choosing sooner and smaller options (SS) increased. In Experiment 2, in the gain situation, after adding the low ego depletion task, there was a marginal significant difference between high and low materialism in the percentage of choosing LL options, *F*(1, 40) = 3.37, *P* = 0.07, η^2^ = 0.08; After adding the high ego depletion task, the percentage of choosing LL options was no difference, *F*(1, 40) = 1.42, *P* > 0.05. In the loss situation, whether in the high ego depletion task [*F*(1, 40) = 2.25, *P* > 0.05) or in the low ego depletion task [*F*(1, 40) = 1.44, *P* > 0.05), there was no difference between high and low materialism in the percentage of choosing LL options, and they both tended to choose SS options. The EEG study showed that in high materialism, there was a significant difference between the high and low ego depletion conditions, and the N1 amplitude induced under the low ego depletion condition was larger than that under the high ego depletion condition. However, there was no significant difference in N1 amplitude between the high and low ego depletion conditions in the low materialism. The amplitude of P2 evoked in the loss situation was larger than that in the gain situation.

**Conclusion:**

In conclusion, Materialism dominated people’s intertemporal choices, and ego depletion affected the intertemporal choice to a certain extent by influencing the subjects’ thinking activities. The COVID-19 epidemic maybe affected intertemporal choice indirectly by acting on materialistic values and subjects’ emotions.

## Introduction

Intertemporal choice is a time-related decision, which refers to the behavior of people weighing and choosing the costs and benefits at different time points (Prelec and Loewenstein, 1991; Frederick et al., 2002; Duan et al., 2017). Intertemporal choice involves two dimensions of time and benefit. This is usually expressed as a small but immediate option (Smaller-Sooner, SS) and an option that is more profitable but needs to be delayed for a period of time (Larger-Later, LL). The two options come in pairs. Intertemporal choice is one of the hot topics in the field of behavioral decision-making in recent decades. It covers all areas of life, such as saving, healthy, environment, economy, and education. Intertemporal choice has an impact not only on people’s happiness, health, and finances, but also on a country’s economic and political prosperity (Reeck et al., 2017; Schonfeld and Chang, 2017).

Most researches on decision-making situations focus on gain and loss situations. The sign effect shows that the individual’s discounting rate is different under gain and loss situations, and the discount rate in the gain situations is larger than that in the loss situations. That is to say, the individual prefers the option with a small benefit and short delay time under the gain situation (Tanaka et al., 2014; Wang et al., 2017; Jiang and Liu, 2021; Li et al., 2022). The research on the sign effect mostly adopts the matching task of fixed current value, which is more likely to produce the sign effect. However, in reality, people are more willing to accept a small loss at the moment and avoid a larger loss in the future, which seems to be more realistic. People also found this phenomenon in subsequent studies. People prefer immediate options in a loss situation than in a gain situation. This phenomenon is called the reverse sign effect (Ortendahl and Fries, 2005; Zhuang et al., 2017; Mahboub-Ahari et al., 2019).

Self-control plays an important role in intertemporal choice. Abundant studies have shown that self-control can also be seen as an ability to resist the temptation of immediate benefits for the sake of larger but delayed rewards (Fujita et al., 2006; Luo et al., 2009, 2012; Guan et al., 2018; Turner et al., 2019). To study the influence of self-control on intertemporal choice, we often use the experimental method of ego depletion.

The concept of ego depletion comes from the self-control resource model (Baumeister and Vohs, 2016; Maranges and Baumeister, 2016). In the self-control resource model, due to the previous self-control behavior task consuming self-control resources, individuals show a shortage of self-control resources in later tasks. This phenomenon is known as the ego depletion effect (Hagger et al., 2010). This model mainly includes three points of view: first, self-control behavior consumes self-control resources, and the former task consumes self-control energy, which will lead to the individual’s worse self-control ability in the subsequent task and affect the task performance. Second, self-control behaviors consume the same kind of resources in the task, but the energy consumption is temporary and can be recovered by certain rest or sleep (Muraven and Baumeister, 2000). Third, individuals differ in the number of self-control resources available and are consistent across different domains (Baumeister, 2002). The success or failure of self-control is determined by the number of self-control resources available in the individual resource pool. The study found that the depletion of individual self-control resources (referred to as ego depletion) will affected their subsequent cognitive and behavioral activities that require self-control participation, and then their individual preferences would be affected (Li et al., 2015). Studies have also shown that, compared to high level of self-control, individuals with low level of self-control were more inclined to choose immediate rewards (Figner et al., 2010; Casey et al., 2011). If individuals still prefer delayed and large money options, it means they have a high level of self-control (Luo et al., 2012).

In addition, researchers generally use money intertemporal choice tasks to study the mechanism of self-control. Individuals need to choose between immediate and small money and delayed and large money. According to the dual processing model, decision-making will consume self-control resources, but this will vary depending on the nature of the task and the processing system involved (Li et al., 2019). When individual self-control resources are sufficient, a more analytical system (system 2) will be adopted for processing to complete the decision-making task. However, the heuristic system (system1) plays a more dominant role when the individual is in a state of ego depletion (Baumeister et al., 2008).

In the field of intertemporal choice, it is found that the value attribute of decision options can influence people’s choices, which is called the magnitude effect (Tayler et al., 2009). However, few researchers pay attention to whether the subject’s monetary values have an impact on intertemporal choice (Yang et al., 2018). With the gradual improvement of people’s living standards, people’s pursuit of material property is more and more direct and intense, and Materialism is mentioned more and more frequently. Materialism refers to having strong material needs and desires, attaching importance to material life style and life philosophy, and taking obtaining material wealth as the goal in life. Excessive materialism values are embodied in the forms of one-sided hedonism, excessive consumerism, radical utilitarianism, and so on, which will cause harm and influence human society, the ecological environment, and human development. At present, COVID-19 has led to a global economic downturn and more uncertainty. People’s economic income has declined, and their confidence and expectation of future income have changed greatly. In this context, paying attention to the influence of materialism on intertemporal choice is helpful to strengthen the education and guidance of reasonable material values for college students.

A large number of ERP studies related to decision-making have shown that N1 and P2, two specific EEG components, can characterize the cognitive mechanism of staged processing of decision-making (McClure et al., 2004; Paynter et al., 2009; Schmidt et al., 2017). N1 is an early negative component in the frontal lobe, which is affected by attention and represents the attention process in the early stage of decision-making. The more attention resources are devoted to the task, the more inclined to use the rational analysis system, and the greater the amplitude of N1 is. Dou et al. (2014) used ERP technology to study the impact of ego depletion on intertemporal choice. The results showed that under the condition of high ego depletion, individuals had a larger discount rate and were more likely to make impulsive decisions. Compared with those with low ego depletion, those with high ego depletion induced a smaller amplitude of the N1 component, that is, lower N1 indicates high consumption of self-control resources, and they could not resist the temptation to choose the SS option, giving up the LL option. However, there are also studies with opposite views. Harris et al. (2013) used ERPs to verify that decreased N1 amplitude implies successful self-control when people can resist immediate temptation. Their behavior showed that they abandoned the SS option and chose the LL option.

It has been found that P2 could constitute a preliminary assessment of the decision-making process related to gain and loss (Gui et al., 2016; Zhao et al., 2018). P2 is a significant positive component in the frontal region after N1, which is related to the attention state, recognition speed and problem difficulty of decision-makers. The slower the recognition speed, the more attention resources and control resources are devoted, the more inclined the individuals are to choose the intuitionistic heuristic strategy for decision-making, and the larger the amplitude of P2 is. Dou et al. (2014) confirmed that the amplitude of the P2 component induced by high ego depletion was larger than that of low ego depletion. But there are also contrary research conclusions. Regarding an intertemporal choice task, compared with a small delayed reward amount and a short delay time, a large delayed reward amount and a long delay time induced larger P2 (Gui et al., 2016; Wu et al., 2016).

The incongruity of the above research conclusions urges us to further explore the neural mechanism of self-control affecting intertemporal choice.

Therefore, this study will explore the influence of materialism and ego depletion on intertemporal choice through two studies. In experiment 1, we used the MVS and the task paradigm of intertemporal choice to study college students, so as to reveal the differences in intertemporal choice between high and low materialism in different situations. In experiment 2, on the basis of experiment 1, ego depletion variables were added. A classic Stroop task was adopted to manipulate ego depletion. And event-related potential technology was introduced to study the changes in intertemporal choice and the differences in brain electrical activity in the high and low materialism at the different levels of ego depletion.

The research logic of these two experiments is progressive. The first experiment is an exploratory experiment on the influence of materialism and situations on intertemporal choice. The second experiment is the research on the neural mechanism of materialism affecting value assessment and self-control affecting resource allocation, which is the in-depth verification of the hypothesis. Accordingly, we propose the hypothesis:

Hypothesis 1: Subjects with high and low materialism have different choices of intertemporal choices in different situations. In the gain situation, subjects with high materialism prefer the delay option.

Hypothesis 2: Different self-control resource depletion affects the intertemporal choice for subjects with different materialism.

Hypothesis 3: The amplitude of N1 evoked by the subjects under different ego depletion conditions was different during the intertemporal choice task, and the amplitude of N1 evoked by the subjects in the low-ego depletion was larger than that in the high ego depletion. The P2 amplitude induced by the intertemporal choice task is different in different situations, and the P2 amplitude induced by the loss situation is larger than that induced by the gain situation.

## Study 1: The effect of materialism on intertemporal choice

The purpose of this experiment is to explore the influence of materialism on intertemporal choice in the situation of gain and loss. The aim is to test hypothesis 1.

### Methods

#### Subjects

A power analysis conducted in G*power (version 3.1.9.2) (Faul et al., 2007) indicated that a minimum of the required total sample size was *N* = 27 to achieve a sufficient power (1-β = 0.95, α = 0.05) with a medium effect size of *f* = 0.25. Based on the consideration of sample loss rate, this study selected 120 college students and tested them with the college Students’ MVS. Rank the total score of the questionnaire. According to the ranking of the total score of the questionnaire, and then according to the principle of a high score and a low score of 27%, 33 subjects (25 women) were finally selected into the high group, namely the high materialism level group, with an average age of 19.12 ± 0.65 years. There were 32 subjects (22 females) in the low materialism group, with an average age of 19.59 ± 0.96 years. All subjects were right-handed, had normal visual acuity or corrected visual acuity, had no relevant experimental experience, and were given certain remuneration after the experiments.

#### Materials

##### Materialistic values scale

The scale for College Students (MVS) developed by Richins and Dawson (1992). The scale consists of 13 items, including three dimensions: success, happiness, and center. The scale adopts the five-point scoring method, with 1 indicating strongly disagree and 5 indicating strongly agree. The higher the total score of the subjects, the higher the level of materialism. The scale has good internal consistency reliability and empirical validity. In this study, the Cronbach’s alpha of this scale was 0.78.

Intertemporal Choice Task. The experimental tasks were similar to those employed by former studies (McClure et al., 2004; Kable and Glimcher, 2007; Xu et al., 2009; Huang et al., 2017; He et al., 2020) and asked the subjects to choose between a small amount and immediate option (SS) and a large amount and delayed option (LL). Referring to the former research results, the delay time of the options was set as two, namely today—half a month later and a today-1 month later, respectively. The difference rate between the two options is 5, 10, 15, 25, 35, 50, 70, and 95%. The intertemporal choice was studied in both gain and loss situations. In the gain (loss) situation, the money in the option represents the amount of gain (loss) and the time represents the time of exchange.

#### Experiment design and procedure

We employed a mixed design of 2(subject type: high materialism level group and low materialism group) × 2(situation: gain and loss) × 2(delay time: half a month and a month). The Subject type was the between-subject variable, and the situation and the delay time were the within-subject variables. And the dependent variables were the percentage of the subjects choosing the delay option (LL) and the response time.

**FIGURE 1:**
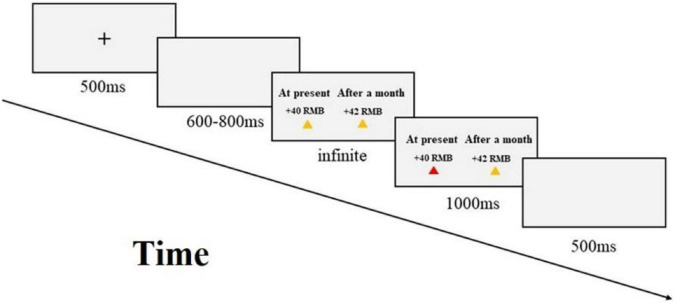
Flow chart of the intertemporal choice task.

All subjects completed the intertemporal choice task experiment on the computer. As shown in Figure 1, first, a black “ + ” appears in the middle of the screen to remind the subject to start the experiment. A random blank screen is then rendered, and intertemporal options are displayed on the screen. On the left side of the screen, there are small and immediate options; on the right side, there are large and delayed options. Subjects need to make a selection response in this interface. Select the left option and press the F button, select the right option and press the J button. After pressing the button, the option is rendered again, but the triangle under the selected option changes from yellow to red. Finally, it passes through an empty screen to the next trial. According to the decision-making situation, the experiment is divided into 2 blocks, 64 trials each. To balance the order effect, the order presented by the two blocks is random between subjects. The “+” sign in the picture indicates the gain, and the “-”sign indicates the loss. Before starting the formal experiment, the subjects will conduct the practice experiment first.

### Results and analysis

#### Manipulation checks

In order to verify the validity of the grouping, an independent sample *t*-test was carried out for the total scores of high and low materialism groups. Results: *t*(63) = 12.48, *p* < 0.01, *Cohen’s d* = 3.05. The total scores of the high materialism group (*M* = 41.33, *SD* = 4.48) and the low materialism group (*M* = 29.22, *SD* = 3.22) were significantly different, and the two groups of subjects do have differences in the level of materialism.

#### The percentage of the subjects choosing the delayed option

**FIGURE 2:**
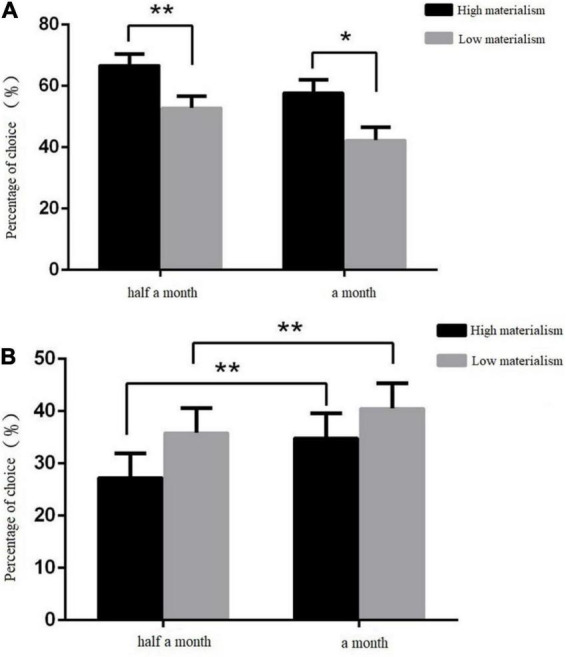
**(A)** The mean and standard deviation of the percentage of subjects choosing the delayed option in the gain situation. *p < 0.05, **p < 0.01, the same below. **(B)** The mean and standard deviation of the percentage of subjects choosing the delayed option in the loss situation.

For the percentage of subjects choosing delay options, repeat measurement ANOVA was conducted for 2 (subject type: high materialism group and low materialism group) × 2(situation: gain and loss) × 2(delay time: half a month and a month). The statistical results showed that the main effect of subject type was not significant. *F*(1, 63) = 1.75, *p* > 0.05. The situation main effect of the situation was significant. *F*(1, 63) = 14.39, *p* < 0.01, η*^2^* = 0.19. In the gain situation (see Figure 2A), the percentage of the subjects choosing the delayed option was significantly higher than that in the loss situation. The main effect of delay time was significant. *F*(1, 63) = 8.26, *p* < 0.01, η*^2^* = 0.12. When the delay time was half a month, the percentage of subjects choosing the delay option was higher than that when the delay time was 1 month. The interaction effects of subject type and situation were significant. *F*(1, 63) = 4.14, *p* < 0.05, η*^2^* = 0.06. The interaction effect between subject type and time was not significant. *F*(1, 63) = 3.12, *p* > 0.05. The interaction between the situation and delay time was significant. *F*(1, 63) = 76.88, *p* < 0.01, η*^2^* = 0.55. The triple interaction effect of situation, delay time and subject type was not significant. *F*(1, 63) = 0.17, *p* > 0.05.

A simple effect test was carried out for the situation and the subject type. The results showed that the percentage of the high materialism group choosing the delayed option was higher than that of the low materialism group in the gain situation. *F*(1, 63) = 7.18, *p* < 0.05, η*^2^* = 0.10. However, in the loss situation, the difference between them was not significant, and there was no statistical significance (*p* > 0.05). A simple effect test of the situation and delay time was performed. The results showed that there was a significant difference between the two delay times in the gain situation, that is, the percentage of subjects choosing delay options when the delay time was half a month was higher than that when the delay time was 1 month. *F*(1, 63) = 67.25, *p* < 0.01, η*^2^* = 0.52. In the loss situation (see Figure 2B), there was a significant difference between the two delay times, that is, the percentage of subjects choosing delay options when the delay time was half a month was less than that when the delay time was 1 month. *F*(1, 63) = 35.98, *p* < 0.01, η*^2^* = 0.36.

#### Response time

For the response time, repeat measurement ANOVA was conducted for 2 (subject type: high materialism group and low materialism group) × 2(situation: gain and loss) × 2(delay time: half a month and a month). The main effect of subject type was not significant. *F*(1, 63) = 0.04, *p* > 0.05.The main effect of the situation is significant. *F*(1, 63) = 4.72, *p* < 0.05, η*^2^* = 0.07. The response time of subjects choosing the delay option in the gain situation was significantly shorter than that in the loss situation. The main effect of delay time was not significant. *F*(1, 63) = 0.16, *p* > 0.05. The interaction effect between the situation and the subject type was not significant. *F*(1, 63) = 0.16, *p* > 0.05. The interaction effect between the delay time and the subject type was not significant. *F*(1, 63) = 3.00, *p* > 0.05. The interaction effect between the situation and delay time was not significant. *F*(1, 63) = 0.59, *p* > 0.05. The triple interaction effect of situation, delay time and subject type was not significant. *F*(1, 63) = 0.18, *p* > 0.05(see Table 1).

**TABLE 1:** Mean and standard deviation of subjects’ choice reaction time in different situations.

Situation	Delay time	Group	
		
		High group (ms)	Low group (ms)
Gain	Half a month	1978.74 ± 204.14	2059.78 ± 207.31
	A month	2136.029 ± 268.29	2046.42 ± 272.45
Loss	Half a month	2240.13 ± 186.83	2480.41 ± 189.73
	A month	2355.97 ± 160.26	2322.68 ± 162.75

### Discussion

Experiment 1 explored the differences between subjects with different levels of materialism in intertemporal choice under different situations and delay time. The results showed that there were differences in intertemporal choice in situations of gain and loss. In the gain situation, the subjects prefer the LL option. However, they tended to choose SS options in loss situations. The hypothesis has been verified. The delay time affected the intertemporal choice. Compared with the delay time of 1 month, the subjects with the delay time of half a month prefer the LL option. High and low materialism have different preferences in intertemporal choice tasks. In the gain situation, the high materialism was more likely to choose the LL option, which was significantly different from the low materialism. In addition, the results of response time showed that the main effect of the situation was significant. The response time of the subjects in the gain situation was significantly shorter than that of the subjects in the loss situation, and the subjects were easier to choose in the gain situation.

## Study 2: ERP study on intertemporal choice by materialism and ego depletion

In Experiment 1, it was found that subjects with high materialistic values preferred to choose LL options in a gain situation, and showed greater patience and long-term planning. However, they tended to choose SS options in loss situations. Previous studies have found that ego depletion promotes impulsive decision-making, preferring to choose SS options (Dou et al., 2014). When individuals with high materialistic values are in a situation of ego depletion, do self-control behaviors fail and preference reversals occur, with more choice of SS options? We will test hypotheses 2 and 3 in Experiment 2.

### Methods

#### Subjects

Use G * power 3.1 software and set effect size *f* = 0.25 (medium size), α = 0.05, 1- β = 0.95, and the sample size required for each group was calculated to be 19. Based on the consideration of the sample loss rate, 110 college students were selected for this study. Subjects were selected in the same way as in Experiment 1. Finally, a total of 21 (12 females) in the high group volunteered to participate in the experiment, that is, the group with high materialism, with an average age of 21.43 ± 2.46 years. A total of 21 subjects (11 women) in the low materialism group volunteered to participate in the experiment, that is, the group with low materialism, with an average age of 20.67 ± 2.01 years. All the subjects in the experiment were right-handed, with no history of brain damage or mental illness, normal naked-eye vision or corrected vision, and no relevant experimental experience.

#### Materials

##### Materialistic values scale

As in Experiment 1, the Materialistic Values Scale (MVS) for College students developed by Richins and Dawson was adopted. Cronbach a of this scale in this study was 0.77.

##### Ego depletion task

The classic Stroop task was used to conduct ego depletion and manipulate the level of ego depletion. A large number of previous studies have found that completion of Stroop tasks will consume individuals’ limited self-control resources, leading to ego depletion. Word-color matching and word-color mismatch were employed. Word-color matching tasks: such as red-colored words and yellow-colored words. Word-color mismatch tasks such as yellow words marked with red and green words marked with yellow. In this study, the Stroop task was divided into four colors: red, yellow, blue, and green, and corresponding words are also divided into four colors: red, yellow, blue, and green. The high ego depletion group performed the word-color mismatch Stroop task with a total of 140 stimuli, including 120 word-color mismatch stimuli and 20 neutral stimuli (e.g., HHH in yellow). The low ego depletion performed the Stroop task with word-color matching, with a total of 140 stimuli, including 120 word-color matching stimuli and 20 neutral stimuli.

##### Intertemporal choice task

On the basis of experiment 1, it was modified. The delay time of choosing the task paradigm was from today to 1 month later, and the other settings were the same as experiment 1.

#### Experiment design and procedure

The experiment adopted a mixed design of 2(subject type: high materialism group and low materialism group) × 2(situation: gain and loss) × 2(ego depletion: high ego depletion and ego depletion). The between-subject variables were the subjects’ type and ego depletion, and the situation was the within-subject variable. And the dependent variables were the percentage of the subjects choosing the delay option and the response time. EEG indexes were N1 and P2 amplitude.

**FIGURE 3:**
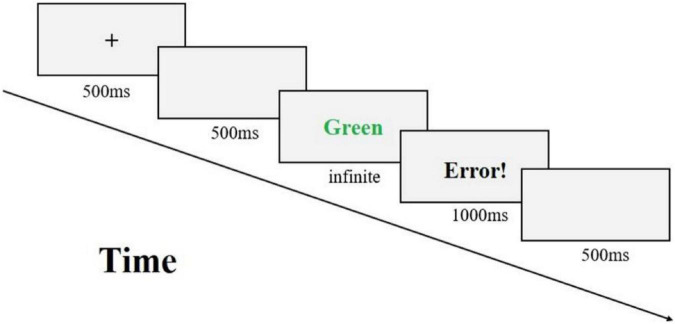
Stroop task flow chart.

The ego depletion task is shown in Figure 3. First, a “ + ” appeared in the middle of the screen to remind the subject to start the experiment. An empty screen is then presented. And then an experimental stimulus is presented on the screen, where the subjects are asked to make a choice response. When seeing red words press “D,” blue words press “F,” yellow words press “J,” and green words press “K.” If the reaction of the subject is correct, it will enter the next trial directly after passing the blank screen. If the reaction of the subject is wrong, the feedback interface will be presented, and an error will appear on the screen! And then the same empty screen into the next trial. In the Stroop task, all the stimulus backgrounds were black, and the size and position of the characters were consistent with a uniform standard, with 140 trials each for the word-color matching task and the word-color mismatch task.

The experimental procedure of the intertemporal choice task was the same as that of experiment 1. Electrical activity was recorded during the intertemporal choice task.

In the formal experiment, subjects first performed Stroop ego depletion task, and answered three retrospective questions after completion. And then performed the intertemporal choice task and recorded brain electrical activity at the same time. The three retrospective questions are designed to examine the ego depletion effect. The three questions include: do you feel tired now after completing the above experiment? (7 points, 1 point no tired, 7 very tired). How much effort did you put into suppressing the effect of literal meaning on color naming? (7 points, 1 not at all, 7 all). After completing the above experiment, how much loss of energy resources do you feel? (7 points, 1 no loss, 7 great loss). In the high materialism group, half of the subjects were given the high ego depletion task, the other half were given the low ego depletion task, and the same was true in the low materialism group.

#### Data recording and analysis

The EEG data was collected by the ANT EEG system. The 64-electrode cap according to the international 10–20 expansion system was used. The sampling frequency was 1,000 Hz. The reference electrode was CPz, and the average value of the bilateral mastoid electrodes was used as reference for the analysis. The band pass is 0.1–35 Hz. During the formal experiment, the resistance between all electrodes and the scalp was less than 5 kΩ. The analysis time course of EEG offline processing is set to −200∼800 ms, that is, 200 ms before the stimulus appears as the baseline level, and 800 ms after the stimulus presentation as the analysis time course.

EEGLAB analysis software was used for offline data processing. The processing steps include loading data, positioning the scalp of the channel, viewing the event value, resetting the reference point, filtering band pass processing, and then segmenting the data, interpolating the bad leads, removing the bad segments, removing the baseline, and removing the electrical eye artifacts by ICA mode for independent component analysis. Then the experimental conditions under the intertemporal choice task were superimposed and averaged, respectively, and finally the data of all subjects were averaged.

### Results and analysis

#### Effectiveness test of experimental manipulation

In order to verify the validity of materialistic grouping, an independent sample *t*-test was carried out for the total scores between high and low materialism groups. Results: *t*(40) = 12.69, *p* < 0.01, *Cohen’s d* = 3.91, high level materialism group (*M* = 41.86, *SD* = 4.27) and low level materialism group (*M* = 27.86, *SD* = 2.71). The results showed that the two groups of subjects do have differences in the level of materialism.

The effectiveness of the ego depletion effect was tested. Three retrospective questions of the high and low ego depletion groups were tested with independent samples *t*-test. The results showed that the high ego depletion group experienced higher fatigue after completing the Stroop task than the low ego depletion group, *t*(40) = 2.95, *p* < 0.05, Cohen’s *d* = 0.91; More effort was put into completing the task, *t*(40) = 3.95, *p* < 0.05, Cohen’s *d* = 1.22; And the task consumed more of their own energy *t*(40) = 5.41, *p* < 0.05, Cohen’s *d* = 1.68. It showed that there was a significant difference between high and low ego depletion groups in self-control resources, and The Stroop task was effective in manipulating ego depletion.

#### Behavioral results

##### The percentage of the subjects choosing the delayed option

In Experiment 2, the descriptive statistics of subjects’ behavior data were shown in Table 2. For the percentage of subjects choosing delay options, repeat measurement ANOVA was conducted for 2(subject type: high materialism group and low materialism group) × 2(situation: gain and loss) × 2(ego depletion: high ego depletion group and low ego depletion group). The statistical results showed that the main effect of subject type was not significant. *F*(1, 40) = 0.10, *p* > 0.05. The main effect of the situation was significant. *F*(1, 40) = 80.76, *p* < 0.01, η*^2^* = 0.68. In the gain situation, the percentage of the subjects choosing the delayed option was significantly higher than that in the loss situation. The main effect of ego depletion was not significant. *F*(1, 40) = 0.16, *p* > 0.05. The interaction effect between the subject type and the situation was not significant. *F*(1, 40) = 0.08, *p* > 0.05. The interaction effect between subject type and ego depletion was not significant. *F*(1, 40) = 0.61, *p* > 0.05. The interaction effect between the situation and ego depletion was not significant. *F*(1, 40) = 0.14, *p* > 0.05. The three interaction effects of situation, ego depletion and subject type were significant. *F*(1, 40) = 5.06, *p* < 0.05, η*^2^* = 0.12.

**TABLE 2:** The percentage of delay options and the mean and standard deviation of response time in different situations.

Situation	Ego depletion	High group		low group	
			
		Percentage (%)	Response time (ms)	Percentage (%)	Response time (ms)
Gain	Low	51.75 ± 4.67	1044.20 ± 346.95	39.36 ± 4.88	1421.28 ± 475.78
	High	43.44 ± 5.39	1187.37 ± 406.47	52.30 ± 5.12	1119.73 ± 410.81
Loss	Low	7.00 ± 3.65	1521.46 ± 542.76	14.91 ± 3.81	1662.40 ± 516.79
	High	13.78 ± 4.21	1525.69 ± 442.71	6.80 ± 3.99	1422.62 ± 532.07

Furthermore, the interaction effects of ego depletion, subjects type and situations were tested. The results showed that under the condition of low ego depletion, the percentage of delay selection in the gain situation was higher than that in loss situation, regardless of the high level materialism group[*F*(1, 40) = 35.82, *p* < 0.01, η*^2^* = 0.49] or low level materialism group[*F*(1, 40) = 9.82, *p* < 0.01, η*^2^* = 0.21]. Under the condition of high ego depletion, the percentage of delay option selected in gain situation was also higher than that in loss situation, regardless of the high level materialism group [*F*(1, 40) = 11.83, *p* < 0.01, η*^2^* = 0.24] or low level materialism group[*F*(1, 40) = 30.91, *p* < 0.01, η*^2^* = 0.45].

A simple and simple effect test was carried out on the interaction effect of situation, ego depletion and subject type. The results showed that in the gain situation and low ego depletion condition, the difference between the high materialism group and low materialism groups in the percentage of delay option selected was marginally significant. *F*(1, 40) = 3.37, *p* = 0.07, η*^2^* = 0.08. In the high ego depletion condition, the high materialism group and low materialism groups were not significant in the percentage of delay options. *F*(1, 40) = 1.42, *p* > 0.05. In the loss situation, no matter in high ego depletion [*F*(1, 40) = 2.25, *p* > 0.05] or low ego depletion [*F*(1, 40) = 1.44, *p* > 0.05], the difference in the percentage of delay options between the high and low level materialism groups was not significant, and they both tended to choose SS options.

##### Response time

For the response time, repeat measurement ANOVA was conducted for 2 (subject: high materialism group and low materialism group) × 2(situation: gain and loss) × 2(ego depletion: high ego depletion group and low ego depletion group). The statistical results showed that the main effect of subject type was not significant. *F*(1, 40) = 0.46, *p* > 0.05. The main effect of the situation was significant. *F*(1, 40) = 25.79, *p* < 0.05, η*^2^* = 0.40. The response time of subjects choosing the delay option in the gain situation was significantly lower than that in the loss situation. The main effect of ego depletion was not significant. *F*(1, 40) = 0.59, *p* > 0.05. The interaction effect between the situation and the subject type was not significant. *F*(1, 40) = 1.03, *p* > 0.05. There was no significant interaction effect between ego depletion and subject type. *F*(1, 40) = 1.81, *p* > 0.05. The interaction effect between situation and ego depletion was not significant. *F*(1, 40) = 0.08, *p* > 0.05. The triple interaction effect of situation, ego depletion and subject type were not significant. *F*(1, 40) = 0.56, *p* > 0.05.

#### ERP results

According to the results of previous studies (Dou et al., 2014; Nie et al., 2018), two EEG components, N1(60∼120 ms) and P2(180∼260 ms) were selected, and the statistical indexes of both time windows were the average amplitude. N1 is an early negative component, affected by attention, representing the attention process in the early decision-making stage. The more attention resources are devoted to the task, the greater the amplitude is. P2 is related to the speed of decision-makers’ attention state recognition and difficulty of problems. The slower an individual is in recognizing problems, the more attention to resources and control resources he devotes, the greater the fluctuation of P2 will be.

(1)N1

**FIGURE 4:**
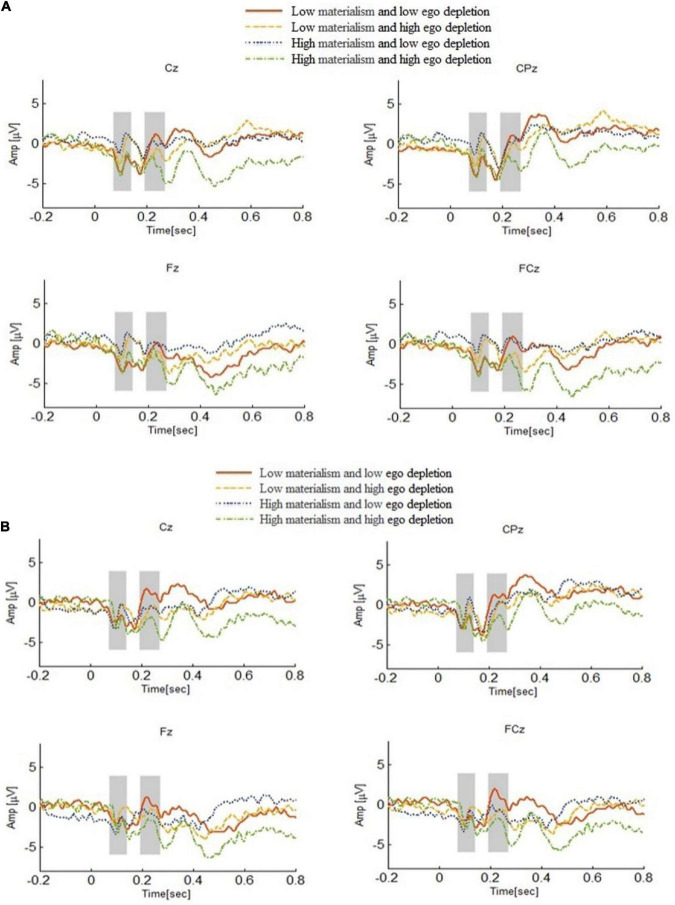
**(A)** ERP wave forms of N1 and P2 components at different ego depletion levels under the gain situation. **(B)** ERP wave forms of N1 and P2 components at different ego depletion levels under the loss situation.

For the average amplitude of N1 (see Figure 4), four factors of repeat measurement ANOVA were conducted for 2(subject type: high materialism group and low materialism group) × 2(situation: gain and loss) × 2(ego depletion: high ego depletion group and low ego depletion group) × 7(electrode points: F3, F4, FC3, F7, F8, FC4, FT7). The results showed that the main effect of the electrode was significant. *F*(1, 40) = 4.46, *p* < 0.01, η*^2^* = 0.11. After comparative analysis, it was found that the amplitude of N1 decreased from the right to the left. The interaction between the situation and the electrode was significant. *F*(1, 40) = 6.99, *p* < 0.01, η*^2^* = 0.16. The interaction effect between ego depletion and subject type was significant. *F*(1, 40) = 7.58, *p* < 0.01, η*^2^* = 0.17. In addition, other main effects and interaction effects were not significant in N1 amplitude.

A simple effect test was carried out for the subject type and ego depletion of the subjects. The results showed that, under the high materialism group, there was a significant difference between high and low ego depletion, that is, the amplitude of N1 induced by low ego depletion was significantly larger than that induced by high ego depletion. *F*(1, 40) = 4.95, *p* < 0.05, η*^2^* = 0.12. However, in the low materialism group, the difference between the two depletion groups was not significant and there was no statistical significance (*p* > 0.05). A simple effect test was conducted on the situation and the electrode, and the results showed that, on electrode F7, the amplitude of N1 induced by the loss situation was significantly greater than that of the gain situation. *F*(1, 40) = 4.40, *p* < 0.05, η*^2^* = 0.10. On electrode FC4 [*F*(1, 40) = 4.25, *p* < 0.05, η*^2^* = 0.10] and F8 [*F*(1, 40) = 9.20, *p* < 0.01, η*^2^* = 0.20], the amplitude of N1 induced by the gain situation was significantly greater than that of the loss situation.

(2)P2

For the average amplitude of P2 (see Figure 4), four factors of repeat measurement ANOVA were conducted for 2(subject type: high materialism group and low materialism group) × 2(situation: gain and loss) × 2(ego depletion: high ego depletion group and low ego depletion group) × 8(electrode points: Fpz, Fp1, Fl, Fp2, F3, AF3, F5, F7). The statistical results showed that the main effect of the situation was significant. *F*(1, 40) = 7.82, *p* < 0.01, η*^2^* = 0.17. The P2 amplitude induced by the loss situation was significantly greater than that induced by the gain situation. The main effect of the electrode was significant. *F*(1, 40) = 10.98, *p* < 0.01, η*^2^* = 0.22. After comparison, it was found that the amplitude of P2 decreased from both sides to the middle. The interaction between the situation and the electrode was significant. *F*(1, 40) = 7.90, *p* < 0.01, η*^2^* = 0.17. Ego depletion and electrode interaction were significant. *F*(1, 40) = 3.06, *p* < 0.01, η*^2^* = 0.08. In addition, other main effects and interaction effects on P2 amplitude were not significant.

The situation and the electrode were further tested for simple effects. The results showed that on electrode Fp2, the amplitude of P2 induced by the gain situation was significantly greater than that induced by the loss situation. On electrodes F3, F7, AF3, F1, and F5, P2 amplitude induced by the loss situation was significantly greater than that induced by the gain situation. A simple effect test was conducted on ego depletion and electrode, and the results showed that the difference was marginally significant in the high and low ego depletion of electrodes F3 (*p* = 0.07), Fp2 (*p* = 0.06), and F1 (*p* = 0.054), while the difference was not significant in the other electrodes.

### Discussion

Experiment 2 discussed the influence of ego depletion and materialism on intertemporal choice, and investigated the behavioral differences and internal neural mechanism of intertemporal choice tasks of subjects with different materialism under situations of gain and loss. The results showed that ego depletion through the stroop task was effective, and the difference in self-control resource depletion affected the choice of intertemporal choice tasks for different materialism. Hypothesis 2 was confirmed. after the ego depletion task was added in Experiment 2, regardless of whether the ego depletion is high or low, the percentage of subjects in the high and low materialism who chose the delay option was greater than that in the loss situation; in the low ego depletion, the subjects with the high materialism group and the low materialism group had a marginal significance in the percentage of choosing delay options. In the high ego depletion, the subjects in the high and low materialism had no significant percentage difference in choosing delay options. in the loss situation, no matter under high or low ego depletion, the percentage difference between the two groups of subjects in choosing the delayed option was not significant, and they mainly chose the small and immediate option.

In the group with high materialism, the amplitude of EEG component N1 induced by the subjects in the low ego depletion was larger than that in the high ego depletion. The P2 amplitude of EEG induced by the intertemporal choice task is different in different situations, and the P2 amplitude induced by the loss situation is larger than that induced by the gain situation. Hypothesis 3 was confirmed.

## General discussion

The research in Experiment 1 found that the interaction between subject type and situation was significant. In the gain situation, the subjects in the high materialism group preferred the delay option. In experiment 2, the three factors interaction effect test also found that, in the gain situation, under the low ego depletion, the percentage difference of choice delay options between the high and low materialism group was marginally significant, while under the high ego depletion, the percentage difference of choice delay options between the high and low materialism group was not significant. In the loss situation, whether in the high ego depletion or low high ego depletion, the results were consistent with the results of Experiment 1. The two groups mainly chose the small and immediate options, and they had no significant difference in the percentage of delay options.

### Reverse sign effect

The reverse sign effect refers to the asymmetry of gain and loss situations. This phenomenon was found in both experiment 1 and experiment 2. Whether in the high materialism group or the low materialism group, as well as under different ego depletion levels, the subjects were more inclined to choose small and immediate options in the loss situation. This phenomenon of negative discount in intertemporal choice refers to a phenomenon that violates the time discount (Sun et al., 2016). This was consistent with the findings of Zhuang et al. (2017).

Under the gain situations, the subjects mostly chose the LL option, indicating that materialistic values played an important role in intertemporal choice, in order to obtain greater benefits. In the loss situations, people prefer the SS option more than that in the gain situations, that is, the discount rate in the loss situations is greater than that in the gain situations, which means that the “reverse sign effect” essentially reflects the role of materialistic values.

The prospect theory (Kahneman and Tversky, 1979) also shows that People have different sensitivities to gain and loss. In intertemporal choice, people are more sensitive to loss of money than they are to gaining money and are more disgusted with loss of money. In the face of loss situations, people are more inclined to reduce their losses, more willing to stop loss in a timely manner, and more willing to choose immediate and small options, leading to a decrease in the percentage of delayed options. Subsequent studies also confirmed this phenomenon (Tang et al., 2017; Zhang et al., 2018).

It also can be explained from the perspective of expected emotions. The waiting process itself is painful and disgusting, so people prefer to have events happen immediately. The research also confirmed that negative emotions can increase the time discount rate of individuals, making them more inclined to short-term options in the loss situations (Liu et al., 2013; Guan et al., 2015; Celeghin et al., 2017; Khosravi et al., 2020).

### Materialism and ego depletion with intertemporal choice

The results of both experiments showed that high materialism subjects prefer to choose large and delayed options in the gain situation. The reason is that materialism is the value of money first. Individuals with high materialism have a stronger desire to obtain and possess money. They focus on obtaining property and define success with money. They have stronger self-control and computing ability when facing intertemporal choice tasks. They adopt rational analytical thinking and are more willing to wait patiently in order to obtain greater benefits. Therefore, they tend to choose the large and delayed options to obtain a greater reward. They may consider the influence of factors such as the attributes of decision options (Mahboub-Ahari et al., 2019), and they tend to choose options with large value and delay options in results (Seaman et al., 2018; Baldassi et al., 2020).

No matter high or low ego depletion, no matter high or low materialism, the percentage of choosing delay options in the gain situation is greater than that in the loss situation, and the difference is significant. This is because materialism deeply determine people’s intertemporal choice behavior.

In experiment 2, under the gain situation, the three-factors interaction test also found that the difference of the delay option percentage was margin significant between the high and low materialism groups under the low ego depletion condition. Under the high ego depletion and the gain situation, there was no significant difference in the percentage of delay choice between the high and low materialism groups. In the loss situation, whether under high ego depletion or low ego depletion, the results were consistent with experiment 1. The two groups chose the small and immediate option as the main choice, and had no significant difference in the percentage of delay options. One of the reasons is that tasks with high ego depletion consume more self-control resources, making them lack self-control and patience. It has a certain impact on the subsequent intertemporal choice tasks and reduces the choice of delayed options. Therefore, under the condition of high ego depletion, there is no significant difference in the percentage of high and low materialism groups choosing delayed options.

Secondly, we speculate that this result was caused by the serious uncertainty of people’s expectations for the future caused by the current COVID-19 epidemic. For human beings, future rewards in intertemporal choices are risky. Uncertainty equals danger, which will lead to great anxiety (MacDonald et al., 2015). Heuristic thinking (system 1) will become dominant, reminding people to take action to ensure safety. This was consistent with the findings of Luo et al. (2014); Guan et al. (2015), and Xia et al. (2017). Their results showed that, negative emotion induced individuals to choose the smaller and immediate rewards. Under the background of the economic downturn and thinking mode caused by the COVID-19 epidemic, people’s time discounts will become larger (Liu et al., 2012). In intertemporal choice, they tend to choose SS options to avoid long-term uncertainty. Li J. et al. (2015) found that those who were more intolerant of uncertainty preferred smaller-sooner gains. Wu et al. (2022) verified the causal relationship between uncertainty and intertemporal choice by showing that participants who feel more uncertain are more likely to choose the SS option. Therefore, the uncertainty caused by the current COVID-19 is also the reason for subjects to choose the SS option.

Thirdly, another important factor that affects intertemporal choice is the characteristics of the decision option itself. Its value or delay time will cause subjects to have different choice tendencies. that is, an increased decision weight on time will potentiate SS choices, while an increased decision weight on amount will lead to LL choices. Although the value of the results generated by delay time is large, its appeal is less than the results obtained immediately (Amasino et al., 2019; Kim and Zauberman, 2019). Therefore, in the loss situation, the two groups of subjects mainly choose SS options.

### Neural mechanism

In the ERP experiment, the early components N1 and P2 showed significant differences in some levels of independent variables, and explained the influence of materialism and ego depletion on intertemporal choice from the perspective of neural mechanism.

In N1 amplitude, it is found that the interaction effect between ego depletion and materialism was significant. N1 is an early negative component in the frontal lobe region with an incubation period of about 100 ms. Affected by attention, it represents the attention process in the early decision-making stage. As pointed out by Blackburn et al. (2012), the more attention resources are devoted to the task, the larger the amplitude is. Compared with the low ego depletion, in the high ego depletion, due to the severe loss of self-control resources, the psychological resources devoted to the processing stage of decision-making are reduced, so N1 amplitude is smaller (Unger and Stahlberg, 2011). In experiment 2, the Stroop task was used to consume individuals’ self-control resources. And the intertemporal choice task was carried out immediately after the depletion. However, their control resources could not be recovered in a short time, so the group with high ego depletion had insufficient self-control resources when carrying out the intertemporal choice task, which would lead to lower N1 amplitude induced by high ego depletion. Besides that, Self-control process has the tendency to enhance the value of delayed rewards (Luo et al., 2009). Inadequate self-control resources will cause subjects to choose SS options. However, in the group with low materialism, the N1 amplitude difference between the two states was not significant, which may be because the subjects in the group with low materialism do not value material goods, and do not devote large psychological resources in the intertemporal choice task related to money.

On P2 amplitude, the main effect of the situation was significant. And the P2 amplitude induced by the loss situation was significantly larger than that induced by the gain situation. P2 is an early positive component with an incubation period of about 200 ms. P2 mainly appears in the frontal lobe of the brain (Carter et al., 2010; Liu and Feng, 2012), which is related to the decision-makers’ attention state, recognition speed, and problem difficulty. The slower an individual is to identify a problem, the more attention to resources and control resources he devotes, the greater the fluctuation of P2 will be (Chen et al., 2017). Gui et al. (2016) ERP results suggested that the P2 component reflected an initial valuation of reward and time delay. In the loss situation, due to people having to choose between two-loss options, and people hate the loss of money, the individual who chooses in the loss situation needs to mobilize more attention resources and self-control resources. The choice in the gain situation is made between two options with positive gains. Compared with the choice in the loss situation, people need to devote less attention and self-control resources, so the P2 amplitude induced in the loss situation is greater than that in the gain situation.

The choice of LL rewards requires more abstract thinking, while SS reward choices are associated with concretization (Smith et al., 2018). Synthesize our research and analysis, we maybe deduce the following logic: Sufficient self-control resources caused by low ego depletion tasks, the gain situation and high materialism individuals tend to rational analysis (system 2) and LL options, showing large N1 and small P2 in EEG components. However, Insufficient self-control resources caused by high ego depletion tasks, the loss situation and all individuals tend to heuristic thinking (system 1) and SS options, showing small N1 and large P2 in EEG components (Extreme single electrode is not considered).

The results of this study expand the interpretation of individual decision-making preferences by intertemporal choice theory. First, the intertemporal choice is not just a pure value calculation around the magnitude of gains and losses or delay time, but a projection of deeper values of decision-makers. It is also helpful to deepen the understanding of the interaction between human economic decision-making and high-level social decision-making.

Secondly, the study confirmed that the “reverse symbol effect,” although it is a departure from the traditional intertemporal choice model, is conducive to improving people’s sense of wellbeing in a positive sense, which is more consistent with the logic of people’s thinking and behavior in reality.

This study also has some limitations. First, this study uses the ratio of delayed options as a statistical indicator for intertemporal choice. Recently, more and more researchers tend to use the discount rate as a statistical indicator for intertemporal choice tasks (Huang et al., 2017). Future research can use the discount rate as a statistical indicator to examine intertemporal choice. Secondly, previous studies have found that individuals’ delayed discounting behavior decreases with age (Bixter and Rogers, 2019), and their sensitivity to loss decreases with age (Kardos et al., 2017). The samples in this study are all from college students, and diversified samples are needed in the future to improve the external validity of this study. Thirdly, as we have seen, regulatory focus and decision-maker role effect exert an important influence on the intertemporal choice (Wang et al., 2019), the role of these two variables will be considered in future research.

## Conclusion

In conclusion, Materialism dominated people’s intertemporal decision-making, Ego depletion affects intertemporal decision-making to a certain extent by influencing the subjects’ thinking activities. The COVID-19 epidemic maybe affected intertemporal choice indirectly by acting on materialistic values and subjects’ emotions.

## Data availability statement

The datasets presented in this study can be found in online repositories (https://osf.io/vp364/?view_only=e3efbfb8376c418bbd5abc258ab93eff).

## Ethics statement

The studies involving human participants were reviewed and approved by the Science and Technology Division of Liaocheng University. All subjects signed and provided written informed consent form for this study.

## Author contributions

YP: investigation, data curation, and writing—original draft preparation. JY: writing—review and editing, formal analysis, visualization, and project administration. LZ: conceptualization, methodology, supervision, and funding acquisition. All authors contributed to the article and approved the submitted version.
